# Supplementary material for: Characteristics of Callus and Cell Suspension Cultures of Highbush Blueberry (Vaccinium corymbosum L.) Cultivated in the Presence of Different Concentrations of 2,4-D and BAP in a Nutrient Medium
Source: Plants (Basel). 2024 Nov 22;13(23):3279. doi: 10.3390/plants13233279 (PMC11644369; doi:10.3390/plants13233279)
Supplement: Supplementary file 1 [file plants-13-03279-s001.zip › plants-3323076-supplementary/Table S1.pdf]

Table S1

Primers used to evaluate gene expression of proteins involved in flavonoid synthesis in *V. corymbosum* cells

**Gene (number by RefSeq)**	**Protein**	**Sequence (5'→3')**	**Amplicon size, bp**
FHT*	flavanone-3β-hydroxylase	F: AACGTCACTGCACTAGCAG<br>R: CTCGTTGCTGAATTCGTTGTAG	100
DFR*	dihydroflavonol reductase	F: CACTGAGTTTAAGGGGATTCCTAAGG<br>R: CCCTTCTCCCTACAAGTGTCAATGG	138
CHI (MH321461.1)	chalcone isomerase	F: GCATTTTCAACCGAGGGGAA<br>R: CGGCGCAAACTCAGACTACAT	188
MYBPA1*	transcription factor	F: CCACCAAAGAAGAGGAGGAC<br>R: CCATTGCCATCGAATTTAGAC	194
PAL (MH321457.1)	phenylalanine ammonia lyase	F: TTCAAAGGTGCGGAAATCGC<br>R: ACCAACCAAGTGGCACTCAT	188
CHS1 (JN654702.1)	chalcone synthase	F: TGCTGAGCGAGTACGGTAAC<br>R: ACAGACTATGGAGCACTGCG	173
LAR (MH321470.1)	leucoanthocyanidin reductase	F: CTGGACCAGCTCACCCTAGT<br>R: GACTCCTCTACCAACCGTCG	161
UBQ3b*	polyubiquitin	F: CCTCCACTTGGTGCTCCGT<br>R: AGATGAGCCTCTGCTGATCCG	130
EF1α*	translation elongation factor 1α	F: AGTTTGCTGAGATCTTGACCAAG<br>R: GTCCCTGACAGCAAACCTTC	175
GAFDH*	Glyceraldehyde phosphate dehydrogenase	F: GGTTATCAATGATAGGTTTGGCA<br>R: CAGTCCTTGCTTGATGGACC	102

* The primers were taken from [29]. For the remaining genes, the primers were designed based on the information provided in the UniProt protein sequence database (https://www.uniprot.org/), NCBI Nucleotide (https://www.ncbi.nlm.nih.gov/nuccore), NCBI Primer Blast (https://www.ncbi.nlm.nih.gov/tools/primer-blast/).
